# Affect School for chronic benign pain patients showed improved alexithymia assessments with TAS-20

**DOI:** 10.1186/1751-0759-4-5

**Published:** 2010-06-04

**Authors:** Eva O Melin, Hans O Thulesius, Bengt A Persson

**Affiliations:** 1 Family Care Centre Strandbjörket, Kronoberg County Council, SE-351 85 Växjö, Sweden; 2 Unit of Research and Development, Box 1223, SE-351 12 Växjö, Sweden; 3 Department of Clinical Sciences Lund, Lund University, Sweden; 4 Department of Clinical Sciences Malmö, Lund University, Sweden; 5 Department of Psychiatry, Kronoberg County Council, PO Box 1223, SE-35112 Växjö, Sweden; 6 Department of Psychology, Växjö University, SE-351 95 Växjö, Sweden

## Abstract

**Background:**

Alexithymia is a disturbance associated with psychosomatic disorders, pain syndromes, and a variety of psychiatric disorders. The Affect School (AS) based on Tomkins Affect Theory is a therapy focusing on innate affects and their physiological expressions, feelings, emotions and scripts. In this pilot study we tried the AS-intervention method in patients with chronic benign pain.

**Methods:**

The AS-intervention, with 8 weekly group sessions and 10 individual sessions, was offered to 59 patients with chronic non-malignant pain at a pain rehabilitation clinic in Sweden 2004-2005. Pre and post intervention assessments were done with the Hospital Anxiety and Depression scale (HAD), the Toronto Alexithymia Scale-20 (TAS-20), the Visual Analogue Scale for pain assessment (VAS-pain), the European Quality of Life health barometer (EQoL) and the Stress and Crisis Inventory-93 (SCI-93). After the group sessions we used Bergdahl's Questionnaire for assessing changes in interpersonal relations, general well-being and evaluation of AS.

**Results:**

The AS intervention was completed by 54 out of 59 (92%) patients. Significant reductions in total TAS-20 post-test scores (p = 0.0006) as well as TAS-20 DIF and DDF factors (Difficulties Identifying Feelings, and Difficulties Describing Feelings) were seen (p = 0.0001, and p = 0.0008) while the EOT factor (Externally Oriented Thinking) did not change. Improvements of HAD-depression scores (p = 0.04), EQoL (p = 0.02) and self-assessed changes in relations to others (p < 0.001) were also seen. After Bonferroni Correction for Multiple Analyses the TAS-20 test score reduction was still significant as well as Bergdahl's test after group sessions. The HAD, EQoL, SCI-93, and VAS-pain scores were not significantly changed. The AS-intervention was ranked high by the participants.

**Conclusions:**

This pilot study involving 59 patients with chronic benign pain indicates that the alexithymia DIF and DDF, as well as depression, social relations and quality of life may be improved by the Affect School therapeutic intervention.

## Background

### Alexithymia

The alexithymia construct includes difficulties to identify and describe feelings, difficulties in distinguishing between feelings and the bodily sensations of emotional arousal, constricted imaginative processes and an externally oriented cognitive style [1]. Sifneos first described the dysfunction in 1967, and in 1972 the term alexithymia was introduced [2]. According to Nemiah and Sifneos a deficit in the capacity for symbolization of emotions results in a variety of manifestations including abnormal physiological reactions, a propensity for impulsive behaviour, discomfort with and avoidance of social relationships, and an impaired capacity for self-care and self-regulation [3]. The limited ability to process emotions cognitively by experiencing them as conscious feelings leads both to amplification of the somatic sensations accompanying emotional arousal, and/or to physical reactions as immediate responses to unpleasant arousal [1]. The autonomous reactions may also be enhanced by physiological dysregulation [4-6].

Alexithymia is commonly found in people suffering from psychosomatic and psychiatric disorders including chronic pain patients (prevalence 35-47%) [7-9], hypochondria, substance abuse, eating disorders [1], panic disorder [10] and depression [1,11-13]. In recent years it is debated whether alexithymia is a stable personality trait [11,14,15], or state dependent [12,13]. Particularly the relation between alexithymia and depression is controversial. If alexithymia is a personality trait this would imply that alexithymia increases the risk of becoming depressed. If alexithymia is state dependant then depression would lead to alexithymic features. In depressive states there are cognitive failures like impairment of memory functions [16]. Thus, the cognitive failure of handling emotions while depressed could be part of a general cognitive impairment.

Interpersonal relationships are difficult to deal with for individuals with alexithymia because of lacking emotional comprehension and expression. Therefore, alexithymia might be associated with reduced social support [17-19], that has been considered a protective factor in determining both the development and prognosis of disease and health problems [20,21]. Many psychotherapists claim that alexithymia is particularly difficult to treat with psychotherapy [22-24]. However, in cognitive behaviour therapy (CBT) for treating depression, the therapy outcome was not hindered by alexithymia [25].

### Tomkins Affect Theory

There are four basic definitions in Tomkins Affect Theory (TAT): Affect, feeling, emotion and script. Affects are the innate, unconscious and genetically pre-programmed biological portions of emotions. Each innate affect has a specific program lasting a few seconds involving face mimicry, body gestures, voice, and autonomous nervous and hormone system physiology [26-30]. The innate affects are enjoyment-joy, interest-excitement, surprise-startle, fear-terror, anger-rage, distress-anguish, shame-humiliation, distaste, dissmell and pain. Awareness of an affect is defined as a feeling [26-30]. Emotions are affects intertwined with memory. A triggered affect evokes memories of earlier situations, relationships and scenes where this affect has been triggered before, and in addition, other affects triggered in the earlier situations will be triggered again in the present situation. An emotion lasts as long as memory continues to trigger the affect. Emotions can be a combination of both unconscious affects and conscious feelings. Scripts are learned patterns to handle emotions. Mood is defined as a persistent state of emotion [26-30]. Affects are important messengers to the self. According to TAT we all have basic drives essential for survival. The primary functions of the innate affects are to regulate these drives. The secondary functions are to regulate other affects [26-30].

### Pain

Pain is defined as an unpleasant sensory and emotional experience associated with actual or potential tissue damage, or described in terms of such damage [31]. According to TAT, pain has qualities typical for both drives and affects. Pain is equal to hunger and other basic drives, while necessary for our survival. Pain seen as a drive can be amplified by affects of distress and fear. Pain seen as an affect can regulate all drives; amplify other affects like distress, anger and fear, and reduce affects of enjoyment and interest. Pain induces impaired functions at many levels that activate shame [26-30].

A definition of Chronic Pain Syndrome (CPS) according to Sanders et al [32] involves

a) Persistent pain of at least three months duration consistent with or significantly out of proportion to physical findings. At least two of the criteria b) to e) should also be present: b) progressive deterioration in ability to function at home, socially, and at work, c) progressive increase in health care utilization (such as repeated physical evaluations, diagnostic tests, requests for pain medications, and invasive medical procedures), d) demonstrated mood disturbance, and e) clinically significant anger and hostility.

Physical Symptom Disorder (PSD) is proposed to replace pain disorder together with other somatoform disorders as a diagnostic entity, whenever one or more physical symptoms are currently present, and not fully explainable by other medical or psychiatric disorders [33]. Medically unexplained symptoms were shown to be closely correlated to the alexithymic features difficulties identifying feelings and difficulties describing feelings, but not to externally oriented thinking [34]. The resolution of unexplained physical symptoms is associated with a short duration of symptoms and few physical symptoms at baseline [35]. Central sensitization is associated with chronic benign pain and leads to a reduction in pain threshold, an amplification of pain responses and a spread of pain sensitivity to non-injured areas [36].

### Purpose of study

The main purpose of this study was to evaluate the psycho-educational method Affect School (AS) for participants with chronic benign pain. Would they accept the AS method and benefit from the intervention? Could it cause any harm? We also wanted to investigate a possible connection between alexithymia and the severity of self-rated anxiety, depression, and stress symptoms. A secondary purpose was to evaluate self rated acquired changes in social relations and general well-being, and participants' subjective feelings about having gone through an overall change.

## Methods

### Affect School

AS is based on TAT [26-30] and was constructed by Armelius and Bergdahl [37-39]. Goals of AS are to identify, differentiate and verbally express emotions, and to identify bodily expressions of affects in order to gain better health [37-40]. The AS method was associated with a significant reduction of self-rated anxiety, somatizing, depression and obsessive-compulsive symptoms in primary care patients [40], and reduction of stress and psychological symptoms in employees with chronic stress [37]. The AS comprises 8 weekly meetings of a 5-7 participants' group with two instructors. Each session has a special theme when one or two innate affects are discussed. The instructors systematically teach participants about the innate affects and their physiological expressions emphasizing that emotions are useful signs of one's inner states. At every session participants are encouraged to recall a specific occasion when they experienced the affect being discussed, and to express in detail how that affect was sensed in body and mind, which means that they are encouraged to tell single-event autobiographical memory (ABM) narratives, which is important in psychotherapy [41-44]. Participants then learn how to link affects with memories and specific situations. They also get general knowledge of affects, feelings, emotions and scripts. After eight group meetings each participant is offered individual Script Analysis (SA) treatment on ten occasions [37-39].

AS instructors involved in this study were one psychologist, one physician, one physiotherapist, one social counsellor, one nurse with psychotherapy training, and one occupational therapist. All instructors had a 40-hour AS training, led by Bergdahl and Persson. The first group had alternating instructors but the rest of the groups had the same two instructors during the whole program. The six instructors and three psychologists - not responsible for the group intervention - were engaged in the SA second part of the AS intervention.

### Subjects

Participants were recruited within patients with chronic benign pain admitted to a Centre of Pain Rehabilitation (CoPR), during the years 2003-2005. All participants fulfilled criteria "a","b" and "c" of the CPS definition [32]. All of them also fulfilled the criteria of PSD [33]. On average, at admittance patients suffered from chronic benign pain for seven years. Fifty five participants (93%) were on full time sick leave or full time temporary disability pension, and 4 (7%) were on part time sick leave or temporary disability pension. All of them had a progressive increase in health care utilization. Thirteen participants (22%) took antidepressant medication at admission. Participants were selected whenever the treatment team considered that they would both benefit from AS, and not influence the group process in any harmful way. All but a few participants received the AS intervention after the usual CoPR rehabilitation program.

### Procedures

Psychiatric symptoms were assessed by means of self-report tests, while somatic diagnoses were taken from medical records. Participants were given five self-report instruments before and after the whole intervention. One self-report instrument was used after the AS but before the SA together with the participants' assessments of the instructors and the group intervention. The group dynamics were observed by the instructors.

#### Self report instruments applied before and after the whole intervention

##### 1) 20-item version of the Toronto Alexithymia Scale, TAS-20

The TAS-20 is a self-report scale developed by Bagby et al [45-48] and is based on three factors: F1: DIF, F2: DDF and F3: EOT. The instrument consists of 20 statements graded from one to five. A sum of 61 points or more indicates alexithymia, a sum of 52-60 indicates an intermediate zone, while 51 points or below indicates non-alexithymia. The validity of the three factor structure has been demonstrated in several translated versions [48-51], including the Swedish version [52]. TAS-20 has been compared to the Modified Beth Israel Hospital Psychosomatic Questionnaire (Modified BIQ) which is an observer scale, and concurrent validity of the two tests was found in different cultures [45,46,51,53]. Also the Bermond-Vorst Alexithymia Questionnaire (BVAQ) has shown internal consistency and concurrent validity with TAS-20 [45].

Reference values for Swedish students of psychology (n = 161) are: mean (SD) = 41.6 (9.2) for the TAS-20 total scores; mean (SD) = 15.1 (4.6) for F1 (DIF); mean (SD) = 11.1 (3.7) for F2 (DDF); mean (SD) = 15.4 (3.8) for F3 (EOT) [52]. Cronbach's alpha internal reliability coefficient for the Swedish version is 0.83 for TAS-20 Global score, 0.79 for DIF, 0.77 for DDF, 0.67 for EOT [52]. The significance of the factor scales has shown divergent results [6,54-56].

##### 2) Hospital Anxiety and Depression Scale, HAD

The HAD-test was constructed as a screening instrument to obtain information about anxiety and depression in patients with somatic complaints in medical wards. Questions about symptoms that could be signs of somatic disease were thus avoided. The test consists of 7 statements reflecting anxiety (HAD-A) and 7 statements reflecting depression (HAD-D). Each statement has four response alternatives with scores from 0 to 3. Maximum score for each seven-item subscale is 21. A sum of 0-7 points for either HAD-A or HAD-D indicates no anxiety or depression, 8-10 points indicates mild anxiety or depression, 11-14 points indicates moderate anxiety or depression, and 15-21 points indicates severe anxiety or depression [57,58]. In the present study scores ≥ 8 points were considered as cut-off for depression and anxiety. In a Swedish general population sample (n = 624) HAD-A mean (SD) was 4.6 (3.7), and HAD-D mean (SD) was 4.0 (3.5) [59]. For all patients admitted to the CoPR within the same 2003-2005 period (N = 414), HAD-D mean (SD) was 9.0 (4.1), and median (iqr) was 9.0 (6.0;12.0). HAD-A mean (SD), in turn was 9.5 (4.8), and median (iqr) was 9.8 (6.0;13.0).

##### 3) Visual Analogue Scale for pain, VAS-pain

The VAS-pain, as used here, is a 100-mm visual analogue scale (VAS) on a horizontal line with the descriptor "no pain" at the left end, and the "worst possible pain" at the right end. Participants are asked to mark on the line the point that they feel represent their current state. Zero means there is no pain and 100 the "worst possible pain" [60]. At the beginning of this investigation the participants estimated their VAS-pain together with a "pain matcher" - an instrument giving the patients a small electrical current through the finger. However many participants refused this "pain matcher"; what made us stop using it, thus increasing the VAS-pain response rate.

##### 4) Modified version of European Quality of Life health barometer, EQoL

The EQoL is a 100-mm VAS assessing health-related quality of life. We used a version placed horizontally, and labelled at the left side "My general health is as bad as possible", and at the right side "My general health is as good as possible" [61].

##### 5) Stress and Crisis Inventory-93, SCI-93

With scores ranging 0-4, the SCI-93 inventory consists of 35 questions about stress symptoms divided into four groups: a) muscular symptoms secondary to the action of the sympathetic portion of the autonomous nervous system; b) other autonomous symptoms; c) hormonal symptoms; and d) two questions about memory and ability to concentrate.

Maximum score is 140. Normal score is 0-25 points, 26-50 points indicate "mild" stress, 51-75 points indicate "moderate" stress, 76 points or more points indicate "severe" stress. Cut off considered in this study was ≥42 points. Reference values for the Swedish normal population are mean (SD) = 27.7 (11.0) for men; and mean (SD) = 30.2 (12.0) for women [62].

#### Assessment after group sessions

##### Bergdahl's Questionnaire

The questionnaire contains 3 statements concerning improved or impaired social relations;

a) close relations, b) working colleagues, c) other people; and two other questions concerning general well-being and overall change. The scale is graded from minus two to plus two, where minus two represents maximal unwanted change, zero represents no change, and plus two the maximal wanted change. The number of participants who estimated positive change was compared to the number who estimated negative changes.

The participants also assessed the instructors and the AS group sessions by responding to 14 statements on a *Likert *scale. Here the scale was graded from one to five, where five represented the optimal degree for 13 statements.

##### Observations made by instructors during group sessions

Observations were made by the instructors about spontaneous complaints, the group process and participant's ability to tell single-event ABM narratives [41].

##### Statistics

Though the means differed little from the medians, the use of median, instead of mean values, was chosen to calculate results of the intervention because of the small sample size. Median follow-up scores were compared to baseline scores by means of the Wilcoxon Signed Ranks Test, which was computed by using SPSS for Windows version 14.0. One Way Anova, Post Hoc Tests, and multiple comparisons Bonferroni (MCB) were made when the correlations between alexithymia and anxiety, depression and stress related symptoms were calculated before intervention. Linear Regression models were adopted to evaluate if improvements on TAS-20-scores and its DIF and DDF factors depended on the improvement in HAD-Depression scores. The self estimated changes in Bergdahl's questionnaire were analyzed with the Binomial Test.

##### Ethical approval

The Regional Ethics Committee at Linköping University approved of conducting this research, Dnr: 203/04, January 25, 2005.

## Results

Fifty-four out of 59 participants (92%) completed the intervention, see Table 1 for age and gender distribution. At baseline, 36% of participants scored 61 or above on the TAS-20; indicating a high prevalence of alexithymia; high scores on mood disturbance such as symptoms of depression, anxiety or a combination of both were noted in 52 (88%) participants; self assessed stress symptoms above a 42 cut-off point were seen in 92% (54) of the participants (Table 1). Participants ranked high the group intervention as well as the instructors (Table 2). Participants who scored higher on the TAS-20 also had significantly higher scores on depression, anxiety and stress symptom, as compared with those with normal scores at baseline (Table 3).

**Table 1:** Baseline characteristics of the 59 participants.

Mean age, years (range)	46 (27-64)
	
	N of participants
Women	52 (88%)
Men	7 (12%)
	
Mood and alexithymia	
Anxiety	47 (80%)
Depression	37 (63%)
Combined anxiety and depression	32 (54%)
No anxiety/depression	7 (12%)
Alexithymia	21 (36%)
Alexithymia, intermediate score	15 (25%)
No alexithymia	23 (39%)
	
Somatic diagnoses:	
Fibromyalgia	25 (42%)
Myofascial syndrome	15 (25%)
Whiplash associated disorder	6 (10%)
Lumbago ischias	4 (7%)
Other pain	9 (15%)

**Table 2:** Participants' assessments of the AS and group instructors. Max score 5 (*optimal score 3).

	Md(iqr)
Affect School as a whole	4(1)
Instructors were warm towards the patients	5(0)
Instructors were interested in the patients	5(0)
Instructors were active	5(1)
Instructors were directing (a lot: 5, a little: 1)	3(1)*
Instructors were competent	5(0)
Instructors were supporting	5(1)
Instructors could help	4(1)
Instructors understood and accepted patients.	5(1)
Instructors told about own experiences	5(1)
Participants appreciated instructors.	5(0)
Participants dared to show feelings	4(1)
Participants and instructors had goals in common	5(2)
Cooperation between Patients and instructors	5(0)

**Table 3:** Correlations between anxiety, depression, stress symptoms and alexithymia. *One Way Anova, Post Hoc Tests, (MCB).

	Non alexithymia Mn(SD)	Non alexithymia Md(iqr)	Alexithymia Mn(SD)	Alexithymia Md(SD)	p-value*
Anxiety score (HAD-A)	7.9(3.7)	9.0(6.5;11)	11.3(3.2)	11(8.5;14)	0.006
					
Depression score (HAD-D)	7.1(3.6)	6(5.5;10.5)	11.2(4.4)	12(8;14)	0.003
					
Stress score	55.2(19.1)	53(45.5;75.5)	74.4(20.3)	74(62;83)	0.006
(SCI-93)					

**Figure 1:**
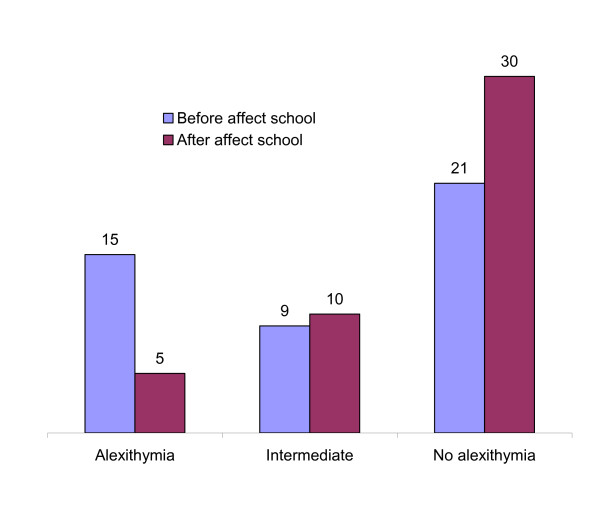
**Number of participants with alexithymia, intermediate values and no alexithymia before and after Affect School intervention (N = 45)**.

The follow-up post-test questionnaires were returned by 46 out of 54 participants (85%) completing the intervention (Additional file 1: Table S1). Significant improvements were seen post intervention in alexithymia, depression, and quality of life scores. After Bonferroni Correction for multiple analyses the change in TAS-20 scores remained significant (p = 0.004). While the DIF and DDF factors of the TAS-20 improved significantly, the EOT did not. After the intervention 10 out of 15 participants (67%) no longer scored above the suggested cut-off point for alexithymia (Figure 1). According to regression analyses, the observed decrease in TAS-20 global score as well as in the DDF score was independent of the diminished depression; whereas 11% of the variance in the DIF score may be explained by changes in depression (Table 4).

**Table 4:** Linear regression analyses of TAS-20 scores and DIF and DDF factors with change in depression scores as independent variable. * = p < 0.05

	TAS-20- difference	DIF-difference	DDF-difference
R	0.17	0.33*	0.13
R Square	0.03	0.11*	0.02

Six out of the 8 non-respondents at follow up had scores above the cut-off point for alexithymia, before intervention. Among the 8 follow-up non-respondents, 3 of them mentioned lack of energy to participate because of divorce, disease or death in the family; 3 other said they disagreed with or simply disliked the instructors; and the other 2 failed to give any reasons for not responding.

**Figure 2:**
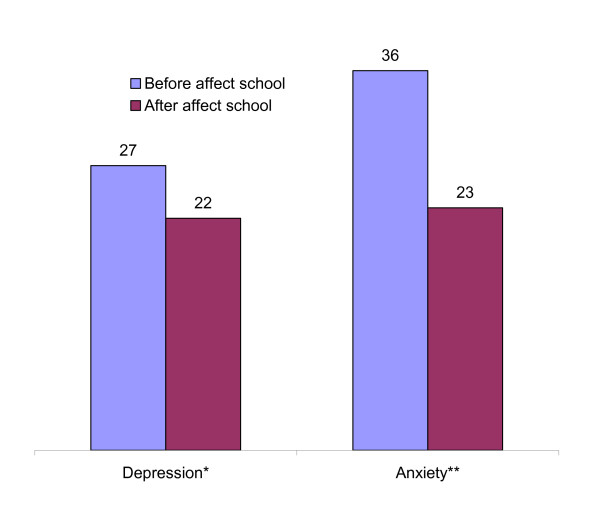
**Number of participants with depression or anxiety before and after Affect School intervention (N = 46)**. Wilcoxon signed Ranks Test: *p = 0.04 (Delta Sum of Ranks 287), **p = 0.11 (Delta Sum of Ranks 253).

After the intervention 5 out of 27 participants (p = 0.04 for changes in median values) no longer scored above the cut-off for depression; and 13 out of 36 (p = 0.11 for changes in median values) no longer scored above the cut-off point for anxiety (Figure 2).

Even after Bonferroni Correction for Multiple Analyses, Bergdahl's test of self-estimated changes in attitudes towards close relations and relationships with other people (working colleagues excluded) improved significantly; as did general well-being and the overall wanted change (Table 5).

**Table 5:** Self assessed changes after termination of group sessions.

	N	Missing	Pos change	Neg change	**p-value**^**#**^
Close relations	48	5	32	1	***
Relation to work colleagues	41	12	1	4	NS
Relations to other people	47	6	23	2	***
General well-being	48	5	22	6	*
Overall change	47	6	36	3	***

^#^After correction for multiple analyses, * = p < 0.05; *** = p < 0.001. NS= Non significant.

### Observations of the group process by instructors

In the first group we had alternating instructors but since group members began to complain, the same instructors were kept throughout the whole AS group intervention for the remaining nine groups. Participants were in general restrained and sceptical during the first session; but a little more talkative during the second session and a strong engagement among participants was noted in the third session. After this third session many participants commented that sessions were too short and eventually many other mentioned they would rather have the group intervention to continue after the eight sessions. Interactions occurred frequently among participants. A bias to tell *general *ABM narratives was noticed, particularly in the beginning [41] and repeated reminders from the instructors were necessary to help participants to tell *single-event *ABM narratives. Many participants had remarkable difficulties to remember any occasion when they had felt the affect joy, and many gave narratives of major traumas when fear was the affect being discussed. Since some of the participants were dominating, while others were shyer and quiet, an important and sometimes difficult task for the instructors was to balance the groups.

## Discussion

### Acceptance of the intervention method

This study includes a group of 59 patients with chronic pain who went through an Affect School (AS) therapeutic intervention. Since 91% of the participants completed the AS and the instructors and the AS received high evaluation scores, we may consider this an adequately accepted method of intervention. We also observed a decrease in scepticism and increased engagement during the course of the intervention, which further suggests good acceptance.

### Alexithymia

The TAS-20 questionnaire has been widely validated and used all over the world, both in clinical situations and in research [45,46,48-52,56]. The 36% prevalence of possible alexithymia pre-intervention in our study was in a range as high as it has been described in other studies of patients suffering from chronic benign pain [7-9]. The lowering of the TAS-20 global score, as well as of the DDF score, was independent of the decrease in depression scores. Theoretically, the connection between alexithymia and depression can be understood both as a trait and as a state-dependent phenomenon; our results don't support the view of alexithymia only as state-dependent. During AS sessions, participants worked through identifying and describing affects and emotions. This corresponds to the DIF and DDF factors of the TAS-20 that showed improved post-test scores, while the factor EOT was unchanged. Based on the results of no changes of EOT even after AS, there is a possibility that EOT is a more trait-prone factor than DIF and DDF. Recent Japanese data show positive correlation between EOT and Constrictive Imaginal Capacities (CIC) [56]. It would be interesting to add therapies like expressive art to the AS in order to evaluate if it also is possible to reduce EOT while achieving improved imaginal capacities.

### Depression and anxiety

As the HAD test is designed to assess mood disorders among patients who seek help for somatic complaints it probably is a good test for a population of chronic pain patients. Other tests, using emotional words and requiring participants to self-rate emotions and moods can give misleading results; particularly among patients with difficulties in identifying emotions. In this study the HAD scores of anxiety and depression pre-intervention were twice as high as for a Swedish general population sample [59]. These baseline scores did not differ between the intervention group and the whole patient population admitted to the CoPR during the same period. Mood disturbances are important factors in CPS, which is also the case for the majority of the patients in our study. After the intervention our patients had a significant decrease in HAD depression scores, which did not remain after Bonferroni correction for multiple analyses. After the intervention more patients also scored below the cut-off for HAD- anxiety, but those with higher scores post intervention had a more pronounced change in their scores, than did those scoring lower. One of the aims of this study was to find out if the AS method could somehow cause any harm. To be aware of one's inner feelings can raise the anxiety levels; which may explain why some of the patients did get higher anxiety scores after the intervention. Maybe a prolonged period of SA and psychological support should be offered to patients with signs of increased mood disturbances. In a previous AS intervention a significant improvement of both anxiety and depression scores was found [40].

### Pain

We have no definite answer to why we did not observe improvements in the pain parameter. The explanation can reside in a variety of problems concerning A) assessment B) emotional factors or C) neurological factors. A) There are several problems with pain assessment. There are only pre and post-intervention scores concerning 38 of the 54 participants who completed the intervention. We do not know if the results would be better or worse if more had participated in the VAS-assessments. As pain is defined as an unpleasant *sensory *and *emotional *experience usually associated with actual or potential tissue damage, or described in terms of such damage [31], we would probably do better having two types of instruments; one instrument assessing the emotional facet of pain and another one just for the sensory part. We tried but failed to assess the sensory part with the so-called pain matcher. The VAS-scale was insufficient for our purpose and we do not know to what extent we were assessing the emotional or sensory part of pain. B) Emotional factors may have influenced the results. For nearly all participants, the rehabilitation period was supposed to be over after the intervention. Maybe participants hoped for more help, thus declaring that they still had significant pain, the actual pain level here somehow representing the suffering with separation from the supportive staff at the rehabilitation centre. Participants may have thought that "all pain is more or less unbearable", and as long as they felt any pain at all, they estimated it as high. The fear of the "pain matcher" might also have influenced the level of pain at follow-up as these assessments were done together initially. C) Neurological factors can also contribute to explain the lack of improvements in pain assessment. Long standing pain can be more or less reversible, but it can also require longer intervention periods due to neurological changes as in central sensitization. In fact it has already been shown that the resolution of physical symptoms depends on the duration and number of symptoms [35]. All our participants had typically suffered for a long time.

### Stress

At baseline 91% of participants had high scores on stress symptoms. The mean scores of SCI-93 were beyond twice as high as the mean score in a general Swedish population sample [62]. Participants within the alexithymia group had significantly higher SCI-93 test scores than did those in the non-alexithymia group. However, the test does not tell us if the symptoms of stress were objectively more severe, or if the tolerance to stress related symptoms was lower in the alexithymia group. Other reports suggest that the autonomous reactions may be exaggerated and prolonged due to physiologic dysregulation [4-6]. The higher stress scores in the alexithymia group may thus simply reflect that their symptoms were more severe. There was no significant change in stress symptoms after the intervention, which is a different result from what Bergdahl et al [37] reported, getting less stress symptoms in employees after the AS intervention.

### Social relations and quality of life

There was a statistically significant improvement of self-rated experiences of close social relationships and relations with others. As previously mentioned, interpersonal relationships are difficult to handle by persons with alexithymia. This can prove to be of major importance while impacting quality of life and health. In the group part of the intervention the participants were trained to be aware of both their own and of other people's emotions. Even though the pain scores did not improve, the EQoL scores, in turn, did. Decreased depression, improved social relations and improved self-understanding may all have a share contributing to the improved quality of life.

### Affect School Method - most important features

The AS method is a structured intervention based on a well defined basic theory, TAT, and combines didactic treatment and emotional-cognitive therapy. The AS can be applied by instructors without prior psychotherapy training. Yet, a 40 hour special training was added to the basic professional competence of the instructors in our study. In every group session each participant was encouraged to tell specific, single event ABM narratives which is regarded as important in other therapies like CBT [41], Emotion-Focused Therapy (EFT) [43], and Client-Centered Therapy (CCT) [44]. The group situation is important as it helps the participants to be aware of own as well as others' emotions; what may, in turn, lead to improved social relationships. The instructors emphasize that affects are important messengers to oneself and all affects are allowed and accepted - an important feature of the AS method.

### Limitations

To assess the effect of an intervention without a comparison group has indeed limitations. Yet, our patients' self defined problem was pain, and not any emotional issue. Thus we did not know, to start with, if they would even accept to participate in 18 sessions talking about affects, feelings and emotions. For that reason we had no comparison group. Patients knew they were participating in a research for a new approach to treatment, and a subject-expectancy effect towards the intervention could thus be expected. It also remains the possibility of an observer-expectancy effect. Yet, scores for alexithymia were reduced more than scores for health related quality of life and depression, while no improvement was observed for pain or anxiety. This outcome specificity suggests a possible effect of the AS intervention over alexithymia, particularly on what concerns its DIF and DDF facets. Although TAS-20 is an instrument well validated in many cultures for both clinical and research situations, our research did nevertheless benefit from a multi-method assessment combining different observer rated as well as self-rated approaches.

## Conclusions

In this pilot study we report of an educational intervention called Affect School directed towards patients with chronic non-malignant pain. The intervention was accepted by most participants. Improvements were seen regarding, depression and general health. Alexithymia features - DIF and DDF - and social relations were significantly improved even after correction for multiple analyses. Pain levels and stress symptoms were not affected.

## Competing interests

EOM was one of the responsible physicians at the CoPR and one of the instructors of the Affect School. The other authors declare that they have no competing interests.

## Authors' contributions

EOM conceived and designed the study, collected and analyzed the data, and drafted the manuscript. BAP and HOT contributed to study design, and in drafting and revising the manuscript. All three authors gave final approval of publication.

## Supplementary Material

Additional file 1 **Table S1**. Scores of self-rated instrument for respondents at both baseline and follow up. Click here for file
