# Supplementary material for: Affect School for chronic benign pain patients showed improved alexithymia assessments with TAS-20
Source: Biopsychosoc Med. 2010 Jun 4;4:5. doi: 10.1186/1751-0759-4-5 (PMC2892428; doi:10.1186/1751-0759-4-5)
Supplement: Additional file 1 — Table S1. Scores of self-rated instrument for respondents at both baseline and follow up. [file 1751-0759-4-5-S1.DOC]

**Additional file 1: Table S1**.

|  | All baseline respon-dents N | Md(iqr) | Respon-dents pre & post inter-vention, N | Patients with improved score  N | Patients with impaired score  N | Baseline  Md(iqr) | Post inter-vention  Md(iqr) | Md diffe-rence | p-value* |
| --- | --- | --- | --- | --- | --- | --- | --- | --- | --- |
| Alexithymia  (TAS-20) | 59 | 55 (47;66) | 45 | 32 | 11 | 54 (45;64) | 47 (39;56) | -6 (-16;2) | 0.0006 |
| F1: DIF | 59 | 22.0 (18.0;27.0) | 45 |  |  | 22.0 (16.0;27.0) | 17.0 (12.5;21.0) | -3.0(-8.0;1.0) | 0.0001 |
| F2: DDF | 59 | 16.0 (11.0;17.0) | 45 |  |  | 16.0 (10.0;17.5) | 13.0 (9.0;15) | -2.0(-4.0;0.0) | 0.0008 |
| F3: EOT | 59 | 19.0 (15.0;22.0) | 45 |  |  | 18.0 (15.0;22.0) | 17.0 (14.0;22.0) | -1.0(-4.0;2.5) | 0.286 |
| Depression  (HAD-D) | 59 | 9 (5;13) | 46 | 26 | 12 | 8.5 (5;11) | 7.0 (5;10) | -1 (-3;1) | 0.036 |
| Anxiety  (HAD-A) | 59 | 10 (8;12) | 46 | 29 | 13 | 9.5 (8;11) | 7.5 (6;10) | -2 (-3;1) | 0.106 |
| General health  (EQoL) | 58 | 40 (30;55) | 45 | 27 | 13 | 40 (30;55) | 50 (35;69) | 10 (8;30) | 0.020 |
| Stress symptoms (SCI-93) | 59 | 68 (47;76) | 46 | 22 | 23 | 62(47;74) | 63.0(47;79 | 1 (-10;8) | 0.892 |
| Pain (VAS) | 57 | 43 (32;62) | 38 | 14 | 21 | 50 (28;4) | 63.5 (27;0) | 4 (-12;20) | 0.156 |
